# Advantages of carbon fibre-reinforced polyetheretherketone over titanium implants in spine SBRT: a phantom evaluation

**DOI:** 10.1007/s13246-025-01587-1

**Published:** 2025-07-21

**Authors:** Simon K. Goodall, Katherine Tonkin, Peter Rampant, Pejman Rowshan Farzad, Martin Ebert

**Affiliations:** 1 School of Physics, Mathematics, and Computing, Faculty of Engineering and Mathematical Sciences, University of Western Australia, Crawley, WA 6009 Australia; 2 GenesisCare, 24 Salvado Road, Wembley, WA 6014 Australia; 3 Centre for Advanced Technologies in Cancer Research (CATCR), Perth, WA 6000 Australia; 4 Department of Radiation Oncology, Sir Charles Gardiner Hospital, Nedlands, WA 6009 Australia; 5 5D Clinics, Nedlands, WA 6009 Australia

**Keywords:** Spine SBRT, CFR-PEEK implants, Titanium implants, Metal artefact reduction (MAR), 3D-printed phantoms

## Abstract

This study evaluates the clinical feasibility of spine stereotactic body radiotherapy (SBRT) in the presence of titanium and carbon fibre-reinforced polyetheretherketone (CFR-PEEK) spinal implants using custom 3D-printed phantoms. The investigation focuses on the dosimetric accuracy, imaging challenges, and achievable localisation precision. Customised 3D-printed phantoms incorporating titanium and CFR-PEEK implants were computed tomography (CT) scanned, with and without metal artefact reduction (MAR) algorithms. Localisation accuracy was tested using Elekta XVI CBCT and Brainlab ExacTrac Dynamic. The dosimetric accuracy of the Monaco treatment planning system (TPS) was assessed under simple geometric conditions and for clinically realistic VMAT plans. Patient-specific quality assurance and phantom-based measurements using ionization chambers and radiochromic film were performed to evaluate delivered dose accuracy. Both Image Guided Radiotherapy (IGRT) systems achieved sub-millimetre localisation accuracy, with maximum deviations of 0.3 mm observed for titanium implants. The Monaco treatment planning system (TPS) demonstrated accurate dose modelling, with deviations < 1% for CFR-PEEK and < 2% for titanium implants in simple homogeneous arrangements. In complex VMAT plan deliveries, dosimetric measurements showed stronger agreement with TPS predictions for CFR-PEEK implants, with deviations < 3%. Titanium-based plans exhibited greater deviations, with localised dose discrepancies exceeding clinical tolerances of 5%. The application of MAR algorithms reduced these discrepancies to < 5%, ensuring clinically acceptable dosimetric accuracy. CFR-PEEK implants enhance clinical workflows due to reduced imaging artefacts and smoother dose distributions, making MAR corrections unnecessary. For titanium implants, MAR is essential to achieve clinically acceptable dosimetric accuracy, highlighting the robustness of CFR-PEEK for spine SBRT.

## Introduction

Stereotactic body radiotherapy (SBRT) of spinal tumours can achieve high rates of local control and effective palliation of symptoms [1]. The steep dose gradients and high precision associated with SBRT are particularly advantageous for spine targets due to the proximity of critical structures, such as the spinal cord, where the accuracy and precision of dose delivery is paramount. However, surgical implants, commonly used for spinal stabilisation in patients with metastatic disease or following surgical intervention, introduce additional challenges to this complex treatment technique.

Titanium has historically been the material of choice for spinal implants due to its strength and biocompatibility [2]. This high-density material can compromise the accuracy of dose calculations and introduce artefacts in both computed tomography (CT) and image-guided radiotherapy (IGRT) systems. Evaluating the accuracy of the implemented clinical Treatment Planning System (TPS) in the presence of these implants is therefore essential to ensure safe and effective treatment delivery.

Previous studies have assessed the accuracy of a range of commercially available TPSs in the presence of titanium implants [3–11]. Liu et al. compared Pinnacle, Eclipse and the Raystation planning systems and reported that clinically relevant metrics generally deviated by less than 2% when implant density overrides were applied or omitted during calculation [9]. However, the study did not confirm absolute agreement with the TPS through measurement, leaving the potential for systematic offsets. In contrast, cadaveric measurements by Grams et al. demonstrated good agreement with film gamma passing rates exceeding 98% when using the Eclipse AAA algorithm, providing further validation [6]. Other studies have shown accuracy levels to be TPS and material specific, highlighting the need for further investigations across other commercial TPSs and implant materials [3, 9].

Recent advancements in spinal stabilisation technology have led to the development of carbon fibre-reinforced polyetheretherketone (CFR-PEEK) implants (CarboFix Orthopedics Ltd., Herzeliya, Israel). These implants are designed to address some of the limitations associated with titanium, featuring a lower density due to their carbon base and an ultrathin (< 0.1 mm) titanium coating. Studies such as that by Nevelsky et al. have examined the dosimetric impact of these materials [12]. Using Monte Carlo simulations (EGSnrc), CT images and measurements through material sheets, Nevelsky reported reduced imaging artefacts and a lesser impact on dose distributions for CFR-PEEK compared to titanium. However, this work used simplified conditions and isolated material geometries, limiting its applicability to complex clinical scenarios.

Müller et al. conducted a treatment planning study comparing titanium and CFR-PEEK implants using Volumetric Modulated Arc Therapy (VMAT) and Intensity Modulated Proton Therapy (IMPT) techniques [11]. They observed minimal differences in the ability to achieve optimal plans between the two implant types. Relative to titanium, dosimetric deviations were reduced for CFR-PEEK implants when Hounsfield Unit (HU) values were varied for VMAT plans, although confirmation through physical measurements was not performed. Others have further investigated the implications for IMPT, but this modality is not discussed further here [5, 10]. Henzen et al. demonstrated feasibility for spine SBRT using the CyberKnife system with carbon-based implants [7]. The CyberKnife system is well designed for SBRT deliveries allowing a largely unrestricted range of beam entry angles. This enables greater freedom during plan creation where minimal interactions with, or complete avoidance of, implants could potentially be achieved. The system is however not as widely available in clinical practice as c-arm linear accelerators (linacs), which more commonly use rotational therapy techniques such as VMAT.

To address the potential challenges associated with clinical workflows using an Elekta c-arm linac and the Monaco TPS, which employs a Monte Carlo dose calculation algorithm, we previously developed a series of custom 3D-printed phantoms [13]. These phantoms replicate lumbar spinal anatomy and incorporate both titanium and CFR-PEEK screws, allowing for detailed investigations of imaging, dose calculation, and treatment delivery under realistic clinical conditions [14].

The aim of this study was to evaluate the clinical feasibility of spine SBRT in the presence of titanium and CFR-PEEK spinal implants using these customised 3D-printed phantoms. Specifically, we sought to assess the accuracy of the Monaco TPS (Elekta AB, Stockholm, Sweden) in modelling the effects of these implants in homogeneous media, the ability of clinically available IGRT systems to localise phantoms containing implants, and the end-to-end dosimetric accuracy achievable in a patient treatment scenario.

## Methods and materials

To enable dose calculation in Monaco v 5.11.02, CT images of all phantoms were acquired using a GE Discovery RT CT scanner (General Electric Healthcare, Chicago, IL, USA) in combination with the clinical spine SABR imaging protocol. This protocol implements a 120 kVp peak tube voltage, GE Smart mA, a slice thickness of 1.25 mm and a 16-bit extended HU range. For the phantoms containing carbon and titanium implants, two reconstructions were generated, one with the GE Metal Artefact Reduction (MAR) applied and one without [15]. No manual overrides were applied to any CT datasets in response to image artefact, however the ionisation chamber inserts were overridden to the density of the immediately surrounding material. These image dataset and phantom combinations will here forth be referred to using the following shorthand;

- Blank: No implant without MAR.
- Carbon MAR: Carbon implants with MAR applied.
- Carbon: Carbon implants without MAR.
- Titanium MAR: Titanium implants with MAR applied.
- Titanium: Titanium implants without MAR.

**Fig. 1:**
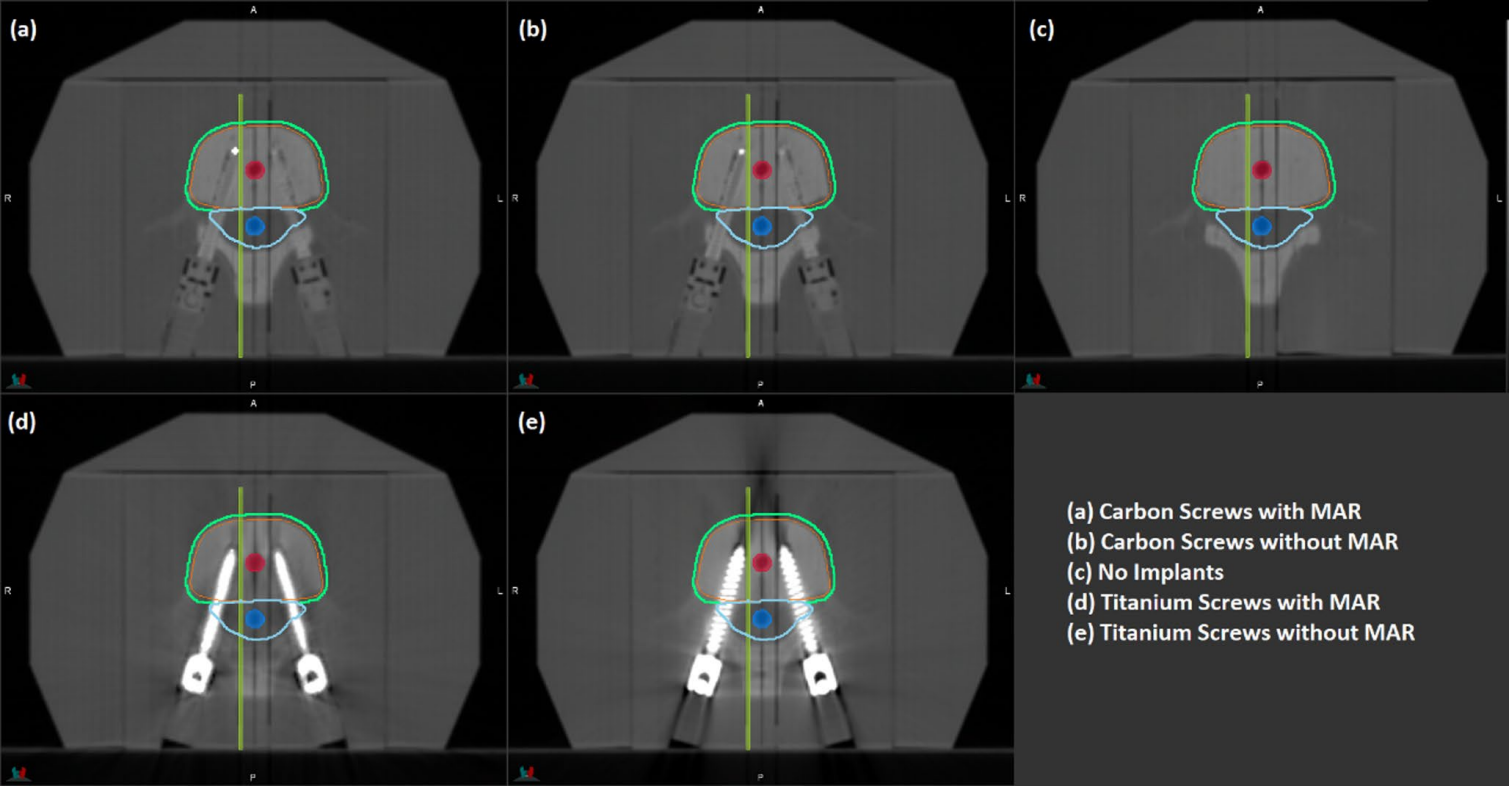
Transverse images through the **a** Carbon MAR, **b** Carbon, **c** Blank, **d** Titanium MAR and **e** Titanium datasets, indicating the location of the bone (red circle), spinal cord (blue circle) and film plane (green line) locations. The PTV volume is shown in green and the spinal canal region shown in aqua

Figure 1 shows a single transverse slice of the three phantoms, indicating the bone and spinal cord ionisation chamber inserts and the location of the film plane [13]. MAR is applied in both images in which inserts are present. The planning target volume (PTV) was created as a 2 mm expansion of the vertebral body consistent with guidance from Cox et al. for single lumbar vertebral level SBRT [16].

### IGRT testing

The ability to position the phantoms using IGRT was evaluated using two imaging systems and two measurements techniques. The Elekta XVI (Elekta AB, Stockholm, Sweden) CBCT and Brainlab ExacTrac Dynamic (EXTD) (Brainlab AG, Munich, Germany) stereoscopic imaging systems were tested using the Carbon and Titanium phantoms along with their reference images. To replicate clinical workflows, reference images were imported into both systems, and regions of interest were defined for subsequent fusions. For the XVI system, the clip box was adjusted to fit within the phantom’s external contour in the left, right, anterior, and posterior directions, and included the vertebra of interest along with half of each adjacent vertebra in the superior-inferior direction. For the EXTD software, the reference images used for matching were restricted to the superior-inferior direction and covered the same vertebral regions.

To evaluate translational error detection, each phantom was positioned sequentially on the Hexapod (Elekta AB, Stockholm, Sweden), a six-degree-of-freedom treatment couch, using treatment room lasers. Seven baseline images were captured at the initial position and compared to the reference image. The average and standard deviation of the baseline images were calculated to assess the imaging system’s repeatability and the baseline offset of the phantom from the reference position. Translational shifts were then introduced using the Hexapod system, with an image captured for each shift. The automatic matching algorithm was used to record the magnitude of the position change relative to the baseline, which was then compared to the induced translation.

To assess the full phantom localisation process, a ball-bearing insert was placed in the bone ionisation chamber holder of each phantom. Treatment plans were generated in the TPS with the isocentre set at the centre of the ball bearing and exported to both imaging systems. The phantom was positioned using the clinical workflow, starting with initial laser alignment followed by translation and rotation corrections based on imaging. After positioning, a 6FFF Winston-Lutz test was performed to determine the ball bearing’s position relative to the linac MV isocentre. This process was repeated separately for both the XVI and EXTD systems.

### TPS comparisons using simple beams in homogeneous media

**Fig. 2:**
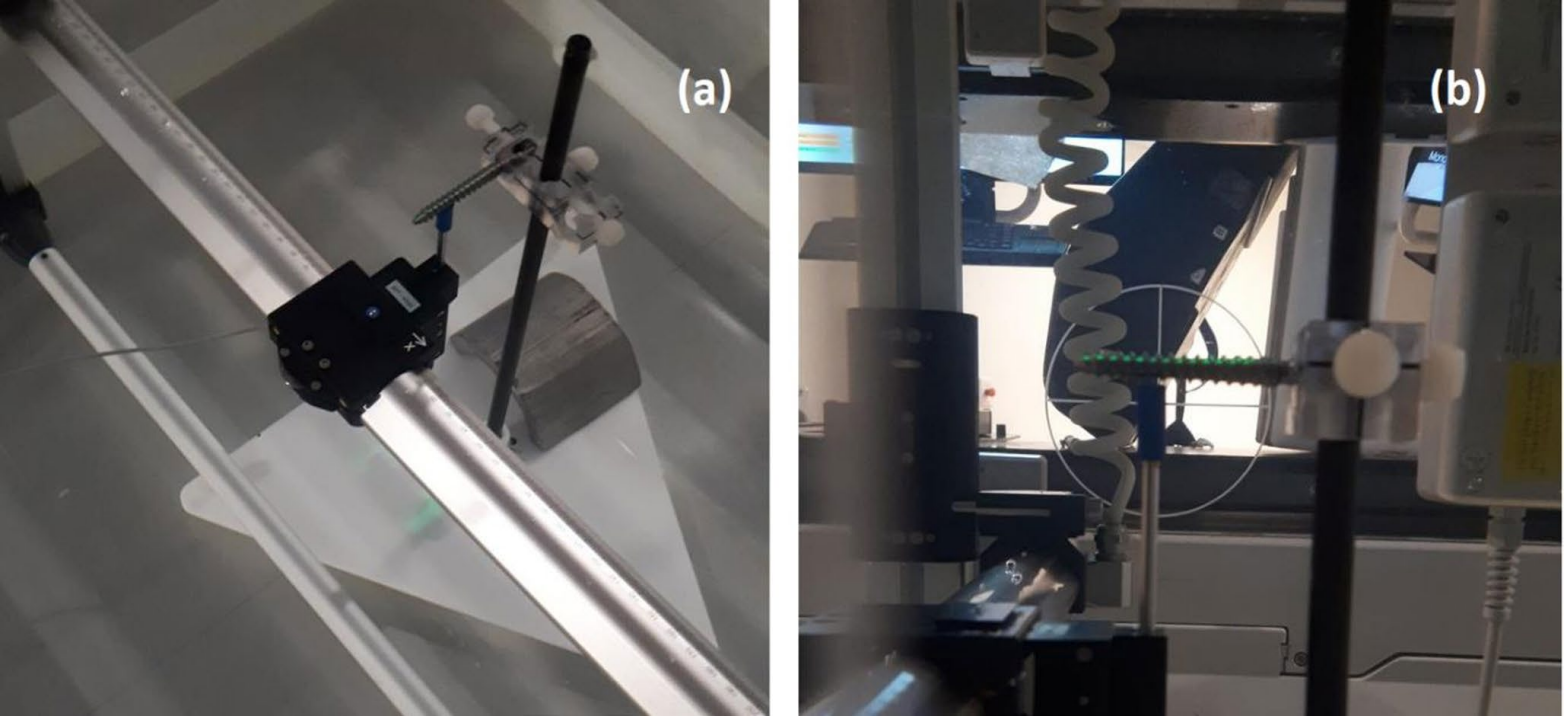
Scanning water tank set-up for measurements of implant attenuation under homogenous conditions

To determine the ability of the Monaco TPS to calculate the dosimetry proximal to implants, measurements and calculations were undertaken in homogenous media for simple static beams. The implants were fixed in place using a custom in-house stand within a 3D scanning water tank. The implants were held at the linac isocentre with their length perpendicular to the axis of gantry rotation. An Electron Field Diode (IBA Dosimetry GmbH, Schwarzenbruck, Germany) was positioned as close as practicable to the implant enabling profile scanning at variable posterior distances as shown in Fig. 2.

Readings were acquired using a 6MV beam, with a 5 × 5 cm treatment field and a 90 cm SSD. To increase captured profile resolution and reduce noise, readings of 50MU per measurement point were captured using a Unidos electrometer across a diode range extending 2 cm on either side of the screw at depths ranging between 1 and 20 mm. The measured profiles containing implants were normalised to the equivalent profile captured with no implant in the beam at a distance of 2 cm off axis to account for any observed drift in diode response with time.

The experimental setup was replicated in the TPS, with dose calculations performed using an isotropic 0.1 cm dose grid resolution and a statistical uncertainty of 0.5% per plan (single field and implant type per plan). In the TPS, screws were modelled as infinitely long cylinders with a single density override applied to each screw. While this simplified model does not replicate the specific manufacturing details of the screws, it provides a realistic approximation within the constraints of clinical treatment planning systems, which are limited by voxel ownership and CT scan resolution. In addition, the investigation focused on dosimetry in the immediate vicinity of the implant, rather than within the implant itself.

To determine the appropriate override value for each implant model, the screws were contoured within the CT scans of the spine phantom, and the mean HU values for these contours were extracted. Based on these values, carbon screws were assigned a relative electron density of 1.300, while titanium screws were assigned a value of 3.975.

The calculated dose planes at the depths of measurement were exported and processed in MATLAB R2022b (Mathworks, Natick, MA) for comparison with the measured profile. A 4 × 4 2D median filter was applied to the exported coronal dose plane to account for statistical noise within the calculation before the isocentric lateral profile was extracted for comparison.

### TPS comparisons using complex beams in realistic inhomogeneous geometries

To evaluate the feasibility of delivering stereotactic spine treatments in the presence of implants, a series of clinical VMAT plans were created meeting the local spine SABR protocol. The key metrics for this protocol require a minimum of 27 Gy to 95% of the target volume with a maximum dose of 35 Gy, while reducing the dose to 0.035 cc of the spinal canal to < 20.0 Gy and 0.1 cc to < 16.3 Gy in three fractions. A total of five treatment plans were developed, one per dataset identified in Section “Methods and materials”.

#### Dose to medium

The Monaco TPS implements a Monte Carlo algorithm to calculate Dose to Medium in Medium D_m,m_. This presents challenges when attempting to measure dose to bone in bone due to the inherent calibration of current detector systems using a dose to water in water formalism [17, 18]. To enable measurement of dose to bone, correction factors for ionisation chambers and film were therefore required [19].

To determine ionisation chamber correction factors, a cross-calibrated Razor Nano Chamber (IBA Dosimetry GmbH, Schwarzenbruck, Germany) was placed in the Blank phantom and localised to isocentre using IGRT. Four 7 × 7 cm square fields, each delivering 500 MU, were delivered from the cardinal gantry angles using a 6 MV beam. The measured dose, corrected for daily output, was calculated using the TRS 398 formalism and compared to the TPS calculated dose. Correction factors for each beam were determined, as the ratio of calculated to measured dose, to achieve agreement with the TPS, and the average of all four factors was calculated. This procedure was repeated with the ionisation chamber placed in the bone and cord insert locations on three separate occasions over one month.

A similar approach was applied for measurements made using radiochromic film. Two phantom modules containing bone material with no implants were used in isolation. Five small pieces of film were sequentially positioned within the bone density region of the phantom, and increasing numbers of MU were delivered using an anterior-posterior static 7 × 7 cm square field. An existing dose-to-water calibration curve was applied to determine measured dose, which was subsequently compared to the dose calculated by the TPS. The resulting ratio was used to calculate a correction factor applied via a masking approach as described by Shaw et al. [19].

This approach assumes an accurate calculation and delivery of a 7 × 7 field to a heterogeneous phantom, introducing additional uncertainty compared to traditional measurements in water. The calculated correction factors were compared to previously published for a PTW microdiamond and EBT3 film in a phantom with similar CT-derived density values, although manufactured from different materials [19].

#### Patient specific QA measurements

The local clinical Patient Specific Quality Assurance (PSQA) processes were completed for each plan. PSQA consisted of an isocentric sagittal measurement using the SNC SRS MapCHECK (Sun Nuclear Corporation (SNC), Melbourne, FL), coupled with two ionisation chamber measurements in a rectangular solid water phantom. The SRS MapCHECK was positioned using IGRT and the ionisation chamber measurements were positioned using treatment room lasers before verification using planar kV images. The first chamber measurement verified the isocentre location (in the centre of the bone PTV) and the second was offset to validate the dose in the centre of the cord structure within the TPS. Both results were reported as a percentage deviation of the isocentre calculated dose.

#### Spine phantom measurements

Clinical plan deliveries were repeated with the ionisation chamber located within the bone insert followed by the cord insert. The measured charges were converted to dose to water and subsequently to dose to bone or dose to cord region tissue using the derived correction factors. Ionisation chamber positioned was completed using treatment room lasers and subsequently verified with planar kV imaging.

Planar dose distributions for each plan were subsequently measured using Gafchromic EBT4 film (Ashland Inc., Wayne, NJ, USA). For each measurement, the detector was positioned appropriately, and the phantom was localised using IGRT. The treatment plan was delivered and the total composite dose recorded. Twenty hours post irradiation, each film was scanned three times using an EPSOM 1100XL scanner and the mean pixel value from the scans was derived. These images were converted to dose to water using eFilmQA (IsoAnalytics Pty. Ltd, Melbourne, Australia) and a binary mask was applied in MATLAB to convert areas corresponding to the phantom’s bone elements to dose-to-bone [19].

## Results

### IGRT testing

The standard deviation values calculated across the seven baseline image sets were less than 0.1 mm and 0.1 degree for all translations and rotations respectively, and for all phantoms. This indicated that the IGRT systems were able to generate consistent images and fusion calculations when imaging in the presence of implants.

**Fig. 3:**
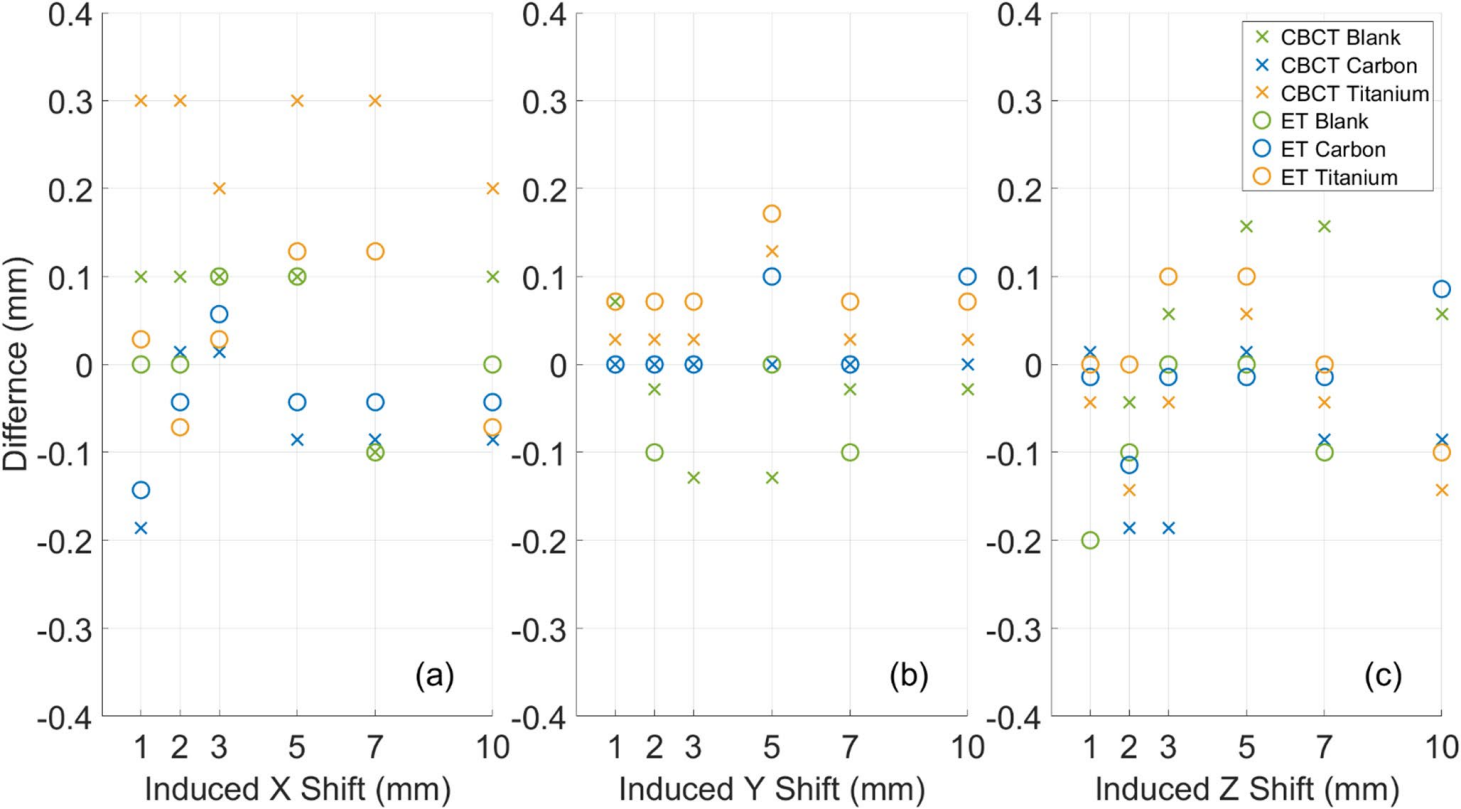
The lateral (**a**), longitudinal (**b**) and vertical (**c**) recorded differences between the applied shift and measured offset, once corrected for initial offsets from baseline position

The difference between the applied Hexapod shift and the measured phantom shift was ≤ 0.3 mm for all measurements across all phantoms, as shown in Fig. 3. This is within the 0.5 mm movement accuracy specification. The largest observed difference was 0.3 mm in a lateral direction for the titanium implant phantom. This result was consistently observed at 0.2–0.3 mm across all moves following the first move from the baseline position, suggesting that the initial Hexapod move may have been slightly larger than expected, or the initial baseline position was calculated with an offset of 0.2–0.3 mm.

Following phantom localisation using the clinical workflow, the ball bearing was found to be within 0.6 mm of the linac MV isocentre in all directions, for all phantoms. This is 0.1 mm greater than the linac tolerance for kV to MV agreement, but remains below the 1 mm which is recommended for localisation accuracy when delivering SBRT treatments [20, 21].

These results, overall, indicate that either IGRT system and associated auto matching algorithms, was able to localise the phantoms with the sub-millimetre accuracy required for subsequent measurements or clinical delivery.

### TPS comparisons using simple beams in homogenous media

**Fig. 4:**
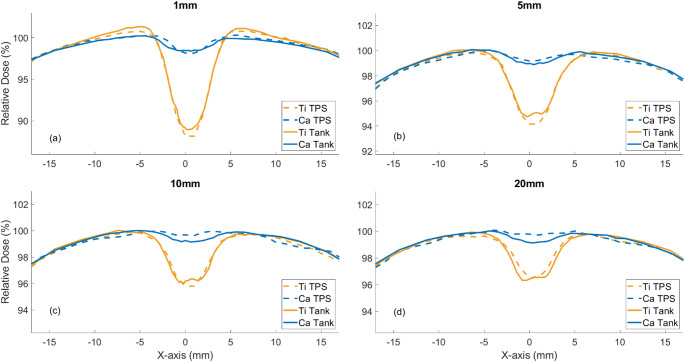
The TPS calculated (dashed lines) and EFD measured (solid lines) relative profiles at distances of 1 mm (**a**), 5 mm (**b**), 10 mm (**c**) and 20 mm (**d**) distance posterior to the screw

Figure 4 displays a comparison between the TPS calculated profiles and the measured profiles at four different distances beyond each screw. The closest possible scanning distance was 1 mm when accounting for the effective point of measurement of the EFD.

The calculated dose showed agreement with the measured profiles to generally < 1%. At 1 mm beyond the titanium screw, a lateral increase in dose relative to the open field profile was observed, due to the increased scatter caused by the titanium implant; however, agreement with the TPS remained within 1%. The lateral dose increases were no longer observable in the measurements at 5 mm.

The measured titanium profiles show a flatter profile than predicted by the TPS at the central axis, which can be attributed to the hollow design of the screw compared to the cylindrical nature of the screw model used in the TPS. The measured carbon profiles show smoother perturbations, lower levels of attenuation, and no lateral dose increase, all of which were well accounted for even by the simplistic model used here.

### TPS comparisons using complex beams in realistic inhomogeneous geometries

#### Dose to medium

For the razor ionisation chamber, correction factors of 0.953 ± 0.001 and 0.995 ± 0.001 were determined for the bone and cord regions of the phantom respectively. A value of 0.940 ± 0.005 was determined for the EBT4 film in bone regions.

#### Patient specific QA measurements

**Fig. 5:**
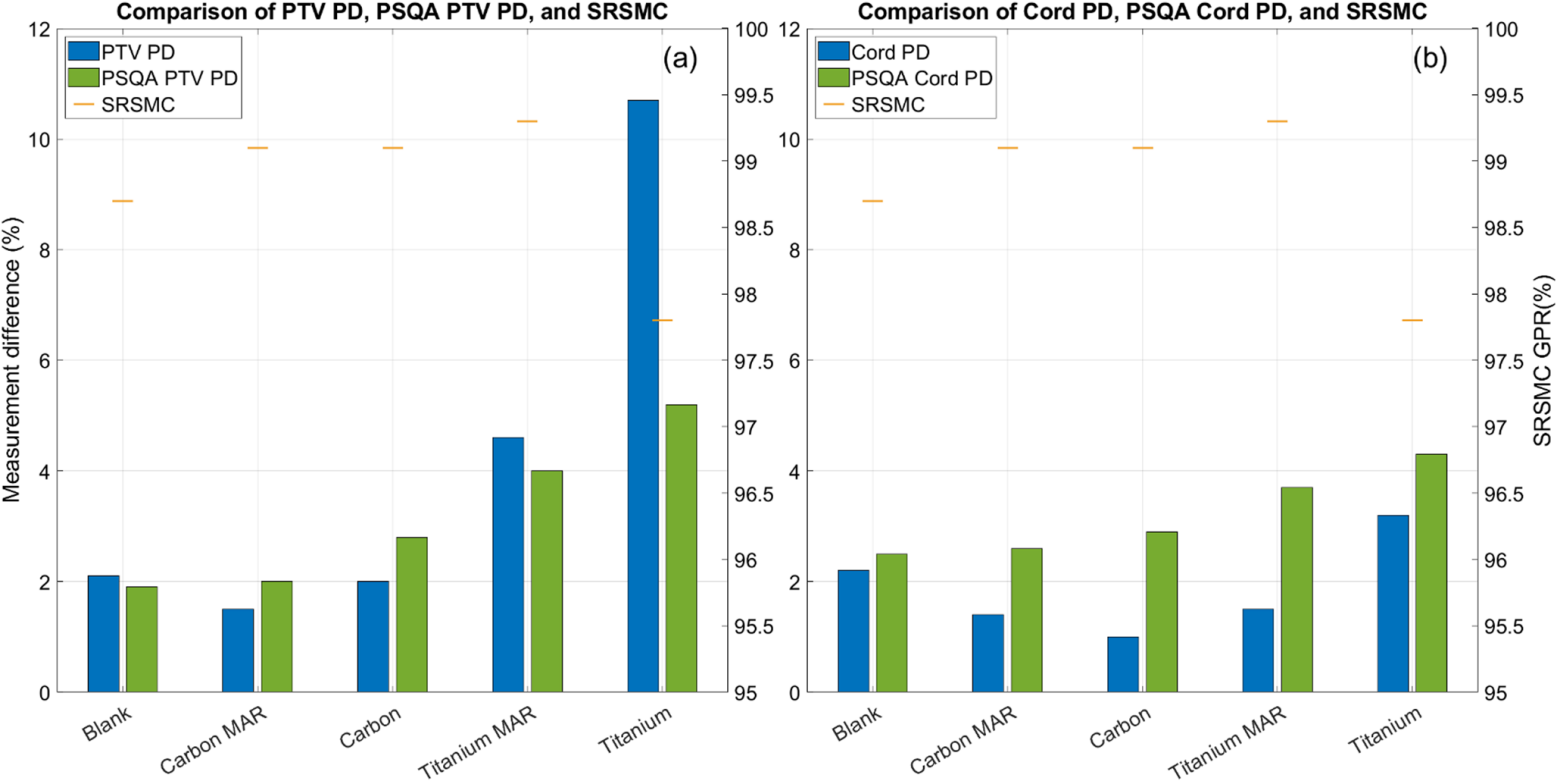
The results of the phantom and PSQA point dose measurements corresponding to the bone insert of the phantoms (**a**) and the spinal cord insert (**b**). SRS MapCHECK gamma pass rates correspond to a single measurement with results replicated on each plot

The results of the PSQA and ionisation chamber measurements are shown in Fig. 5. All SRS MapCHECK gamma pass rates (GPR) using criteria of (3%, 1 mm) were above the 95% clinical tolerance implemented locally. The Titanium dataset beam showed the lowest GPR; however, this was still 97.8% and well within the local tolerance. Ionisation chamber measurements were systematically higher than expected but remained within the in-house tolerance of ± 5%.

#### Spine phantom measurements

The ionisation chamber measurement results are shown in Fig. 5 alongside the corresponding PSQA results. All measurements were within the local ± 5% tolerance excluding those within the in PTV for the Titanium dataset.

In general, the spine phantom results align well with the PSQA ion chamber measurements, showing differences of less than 0.5% in the PTV (excluding the Titanium dataset) and less than 2.5% in the cord. Measurements within the cord showed better agreement within the phantom compared to the PSQA measurements, likely due to the high dose gradients and the IGRT positioning, which was used only in combination with the spine phantom.

**Fig. 6:**
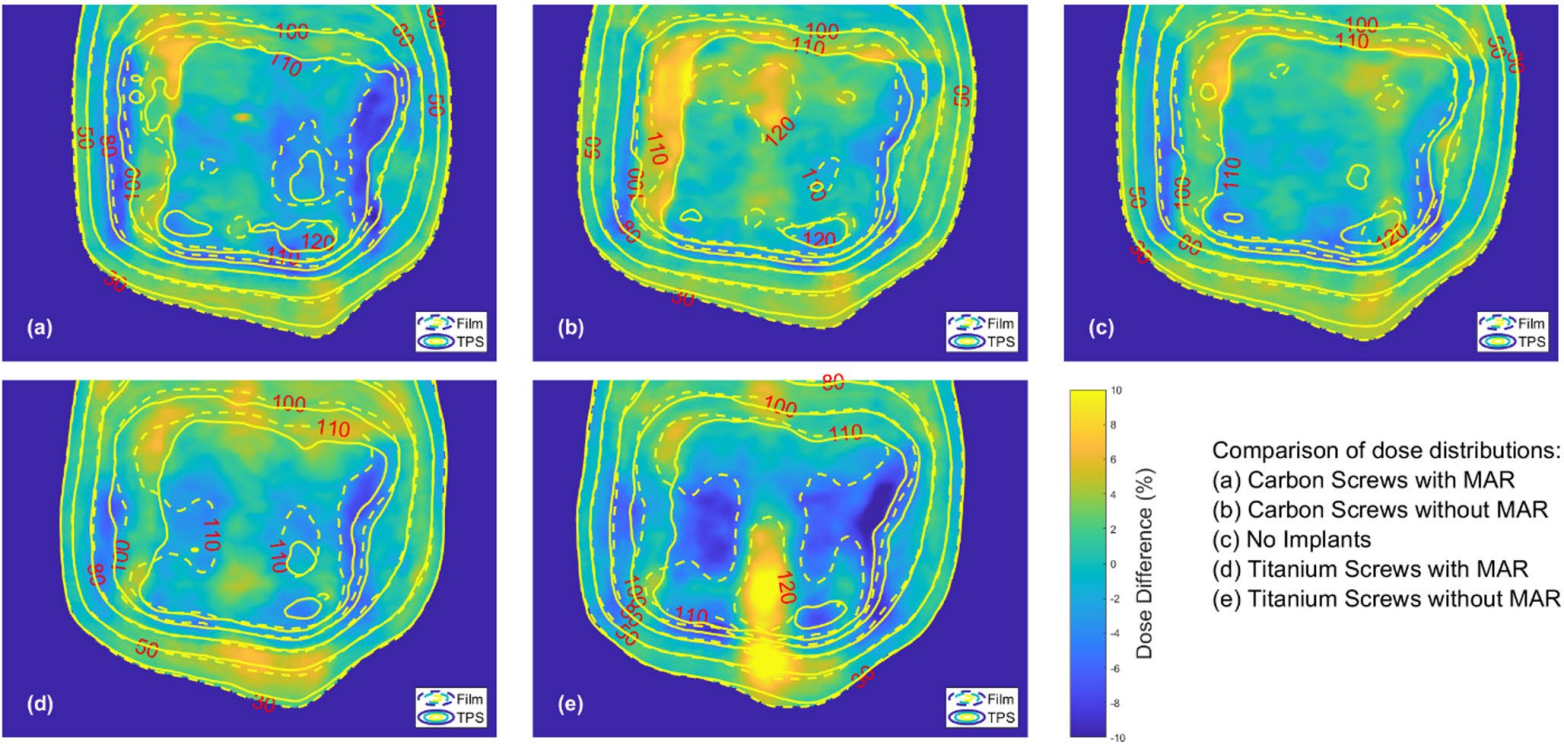
The local dose differences between the TPS calculated and EBT4 measured sagittal dose planes. Each plots displays difference for a given plan and dataset, Carbon MAR (**a**), Carbon (**b**), Blank (**c**), Titanium MAR (**d**) and Titanium (**e**)

Figure 6 shows the measured sagittal film planes for all beams. All measured planes show some areas of dosimetric deviation which highlight the uncertainties in the process, including routine film dosimetry limitations, coupled with dose to medium challenges, particularly in regions of transition from bone to tissue equivalence [19]. The dose differences displayed are local dose differences, due to the focus of this work on dosimetry in proximity to the implants. As a result, the areas of steep dose gradients surrounding the target show exaggerated discrepancies due to the lack of distance-to-agreement metrics.

Of note are the relatively uniform differences between measured and calculated dosimetry from the Blank dataset (c) compared to the other four measurements in which the screw locations can be identified as areas of reduced agreement with the TPS. The Carbon MAR (a) also shows a good level of agreement within the central PTV region. The location at which the tip of the screw is within immediate proximity to the film can be observed as a small erroneous region showing deviations of the order 5–6%. At this point the TPS predicts an increased dose which is not reflected in the measurement.

For the Carbon dataset (b), some differences are apparent in the region shadowed by the screw, running from top to bottom in the images within Fig. 6b. In general, the discrepancy along the length of the screw is within the range of 2–3% which is elevated in relation to the immediately surrounding dose differences, but within the uncertainty of the measurement process. Again, a larger error, in the range 6–7% is observed at the location where the screw is in immediate proximity to the film.

In both the Titanium MAR (d) and Titanium (e) planes the location of the titanium screw is apparent with increased dose measured along the full length of the screw relative to the TPS calculation. In both planes there is additionally underdosing in the areas of the PTV lateral to the implant location. For the Titanium MAR dataset, the dose measured along the length of the screw was of the order 2–3% higher than expected and the regions lateral to the screw were 3–4% lower than calculated. Without MAR these discrepancies increased to 10–12% along the screw’s length and 6–7% within the lateral regions.

## Discussion

This study evaluated the feasibility of spine stereotactic body radiotherapy (SBRT) in the presence of titanium and CFR-PEEK spinal implants using customised 3D-printed phantoms [13]. The results demonstrate that clinically achievable dose accuracy is possible, even in the challenging presence of high-density implants, provided a MAR algorithm is used.

The results from this study demonstrate that the presence of titanium and carbon spinal implants did not notably compromise the localisation accuracy of the IGRT systems evaluated. Both XVI and EXTD achieved sub-millimetre accuracy in detecting induced translational shifts, with no difference in performance between the Titanium, Carbon or Blank phantoms and their associated datasets. Specifically, the maximum observed deviation of 0.3 mm for titanium implants was within the Hexapod movement specification of 0.5 mm. This suggests that the higher density and imaging artefacts associated with titanium did not meaningfully degrade the system’s ability to localise the phantom [7].

These findings highlight the resilience of modern IGRT systems in managing the challenges posed by spinal implants and affirm their capability to maintain clinical alignment accuracy, regardless of implant type. Due to the stereoscopic nature of the EXTD x-ray imaging system, reduced artefacts were observed in the captured images compared to XVI. However, it is important to note that these images must still be registered to CT images containing some level of artefact, even when MAR is used. While the EXTD images could therefore be easier to assess by the treatment team, the fusion algorithm is still required to overcome variations in artefact levels between online and reference datasets.

Under the conditions of a simple beam in homogeneous media, titanium implants caused attenuation exceeding 10% locally and increased lateral scatter at the implant edges as shown in Fig. 4. In contrast, carbon implants exhibited less than 2% attenuation and no observable lateral scatter effects. These measurements are broadly inline with the calculations of Nevelsky et al. when considering the experimental uncertainties [12].

The accuracy of the Monaco TPS in modelling these effects was within an acceptable level for clinical practice, with deviations generally less than 1%. The Monte Carlo algorithm also predicted increases in lateral dose, demonstrating its robustness in accounting for complex material heterogeneities. Central axis deviations were likely due to the simplified TPS screw model but remained within 2%. While the TPS adequately accounted for the presence of either implant type, the reduced magnitude and complexity of the dose perturbations caused by CFR-PEEK implants make them more favourable for clinical practice and ease of TPS modelling. A current limitation of the Monaco TPS is its inability to manually assign material types, which could potentially further reduce dosimetric uncertainty, particularly in regions containing high-density implants.

The PSQA results revealed notable differences in performance between Titanium and Carbon plans despite recalculation on a homogeneous phantom. This highlights the impact of metal-induced perturbations and the associated challenges in achieving target coverage during plan creation. In contrast, the reduced perturbation effects of carbon implants facilitated agreement with calculated doses consistent with measurement for the Blank phantom. This reflects their advantage in minimising perturbations, which can be more easily and accurately accounted for during inverse optimisation. The ionisation chamber measurements all recorded higher than calculated doses, indicating a systematic offset in the TPS modelling or linac performance rather than a phantom specific issue.

The tolerances applied in this study were greater than the 2–3% recommended for Intensity Modulated Radiotherapy (IMRT) to account for the steeper dose gradients associated with SBRT, both within the target and adjacent areas [22]. If more strict tolerances had been applied, the two plans generated on the Titanium and Titanium MAR datasets would have been deemed not suitable for treatment and required replanning. Other studies have explored methods for generating optimal plans in the presence of titanium implants [23], which may have improved both the PSQA results and the level of agreement when delivering plans to phantoms. These techniques were not evaluated here; however, it was effectively demonstrated that they were not necessary for the Carbon or Carbon MAR datasets.

In clinical practice, some centres may choose to contour metallic implants and the associated regions of artefact and override their densities to approximate the true values. Depending on the accuracy of the contouring process and the reliability of the assigned densities, this strategy could be expected to reduce inaccuracies and improve the agreement between planned and measured doses. If implemented for the Titanium dataset, this approach may improve the dosimetric results towards those observed for the Titanium MAR datasets in this study.

The continued passing rates of the treatment plans, as determined using the 3%/1 mm gamma analysis criteria and 95% tolerance, highlight the need to qualitatively review the measured dose distribution alongside the numerical gamma score. This is especially important as localised areas of failure were clearly demonstrated to occur in the regions near the implant during PSQA.

Correlation was observed between ionisation chamber measurements within the homogeneous PSQA and the inhomogeneous phantoms, following the application of the derived dose to medium correction factors. The bone correction factors were comparable to those published by Shaw et al. factors of 0.962 for the microdiamond detector and 0.953 for EBT3 film [19]. The correction factor determined for the cord location reflects the uncertainty in factor derivation and the variation in density between the 3D printed material (~ 1.03) and water (1.00). As different detectors were assessed by Shaw et al. [19], the in-house determined corrections factors were applied during subsequent measurements.

Measurements in the phantom’s bone insert, relative to planned doses, were within acceptable tolerances for the Blank and Carbon datasets. The Titanium dataset results showed unacceptably high disagreements, with deviations exceeding clinical tolerances. The application of MAR (Titanium MAR dataset) significantly improved agreement, reducing discrepancies to within acceptable limits [24]. This improvement was driven by the increased precision in determining the true size, shape and location of the titanium within the phantom. For the Carbon datasets, the reduced image artefact allowed for accurate determination of the implant, even without MAR, demonstrating improved compatibility with inverse planning processes [7, 12].

Ionisation chamber measurements in the phantom spinal cord region showed improved agreement with the TPS when compared to PSQA results. This is attributed to the use of IGRT for phantom deliveries, which were not possible during PSQA. Importantly, even for the Titanium dataset, the doses within the spinal cord insert did not exceed a 5% global difference with the TPS. The Titanium dataset was, however, the only phantom to show a discrepancy of greater than 3%, which is 0.9% greater than the Blank phantom.

The increased distance between the screw and point of measurement in the clinically representative arrangement is the primary reason for maintaining an adequate level of agreement. This distance reduces the relative contribution to the composite dose, of fluence which has traversed an implant material, or is within the region of increased lateral scatter. A similar result was observed by Byrne et al., when dosimetric errors reduced from greater than 10% to under 5% when moving from a single beam to arc based delivery [3].

It is important to note the PTV used in this study consisted of the vertebra only, while targets including the full bone region are treated under some clinical protocols [25]. The positioning of the implants relative to the cord insert within the phantom replicate specific patient anatomy. Nonetheless, the distances from the implants to the spinal cord or canal will vary for every patient and vertebra. This distance should be evaluated to determine the applicability of these results to new patients, particularly for small vertebra within the cervical or upper thoracic region. Future investigations could extend this work by evaluating alternative PTV arrangements, including whole-vertebra targets or multiple dose level treatments and a greater range of treatment plans.

Film measurements provided additional spatial detail regarding localised areas of discrepancy, although with reduced absolute dose certainty compared to ionisation chamber measurements. For the Titanium dataset, deviations of 10–12% were observed along the length of the screw, accompanied by underdosing in regions lateral to the implant. These discrepancies likely stem from the limitations in defining the true size and location of the titanium screw in the reference imaging used for treatment planning. Such dosimetric errors are concerning in the context of both local control and vertebral stabilisation post-treatment. With the application of MAR, discrepancies in the shadow of the screw were reduced to 2–3%, and the lateral underdosing was reduced to 3–4%. While these differences fall within the commonly accepted clinical tolerance of 5%, they underscore the challenges faced by the TPS in accurately managing the combined effects of attenuation and lateral scatter. These relatively complex and undulating fluence patterns should also be considered in the essential need for high accuracy localisation of the patient. Small positional offsets could quickly lead to under or overdosing of areas due to small offsets of the implant from the intended position.

In contrast to results for the titanium screws, the Carbon plans exhibited smoother dose distributions, with deviations remaining below 3% throughout and no sharp changes between under- and overdosed regions. The application of MAR further improved agreement between the TPS calculations and measured doses, although the benefits were less pronounced than those observed for the Titanium datasets. The reduced perturbation effects and less modulated dose distributions generated for the Carbon and Carbon MAR datasets also offers increased robustness against small geometric positional variations. This characteristic further reinforces the clinical advantages of carbon-based implants in a spine SBRT clinical workflow.

## Conclusions

This study evaluated the dosimetric and imaging impacts of titanium and CFR-PEEK spinal implants on the clinical feasibility of spine SBRT using customised 3D-printed phantoms [13]. The findings demonstrate that while titanium implants present notable challenges due to high-density artefacts and dose perturbations, these can be mitigated to acceptable clinical levels with the application of MAR algorithms. In contrast, CFR-PEEK implants exhibited reduced imaging artefacts and dose perturbations, enabling more accurate treatment planning and delivery, even in the absence of MAR corrections.

Both XVI and EXTD IGRT systems achieved sub-millimetre localisation accuracy, highlighting the resilience of modern imaging systems in managing implant-induced artefacts. The Monaco TPS demonstrated high accuracy in modelling the effects of both implant types within homogeneous geometries and regular beam arrangements. The smoother dose distributions and reduced perturbation effects associated with CFR-PEEK implants offer additional clinical advantages, including greater robustness against geometric uncertainties in defining implant size, shape and location within the treatment planning images, as demonstrated during the end-to-end deliveries.

Spine SBRT planned in Monaco is feasible for patients with CFR-PEEK implants without a requirement for MAR corrections, however they can still provide benefit if available. SBRT can be feasible for those with titanium implants if MAR corrections are applied during the capture of planning datasets. If these results are to be generalised to patient treatments, it is essential to account for factors such as anatomical variation and the patient-specific distance between implants and the spinal cord to ensure treatment safety and efficacy.
